# Global trends in open access publication and open data

**DOI:** 10.1002/acm2.13140

**Published:** 2020-12-28

**Authors:** Michael Mills

**Affiliations:** ^1^ Department of Radiation Oncology University of Louisville Louisville KY USA

This Editorial will summarize some of the recent tendencies of publication explored in a recent Wiley Society Newsletter on the open access movement: http://s1133198723.t.en25.com/e/es?s=1133198723&e=6599750&elqTrackId=be52ad97a9d24b6c8db9974cd2051faf&elq=fef810d97dae4c9c9b098792bf9de575&elqaid=48002&elqat=1 . As it turns out, in a recent survey about Society Publications, Wiley determined that no‐cost or open access to Society content is the top desire for most researchers. They also found that making journal articles more accessible to nonacademic audiences, greater transparency around peer review, and improving how we measure the impact of research are also highly important. https://www.wiley.com/network/societyleaders/member‐engagement/members‐say‐open‐data‐is‐more‐important‐now‐than‐it‐was‐12‐months‐ago?elq_mid=48002&elq_cid=12309687&utm_campaign=30355&utm_source=eloquaEmail&utm_medium=email&utm_content=Email%206‐RC‐SOCM‐MS‐XX‐Global‐W26M4‐October%20Newsletter.

While three‐quarters of members are mostly satisfied with the access to society content that they personally receive as members, only a little more than half are happy with their society’s engagement with open access publishing.

Publishing open access is particularly important for recruiting newcomers to your field: 85% of students and 75% of researchers with less than five years of experience told us that societies need to do more OA publishing. Researchers in the USA show the least demand for more OA (61%), but if your society is interested in growing membership in Africa or the Middle East, try emphasizing open access in your outreach: 84% in Africa and 82% in the Middle East want more society open access opportunities.

More researchers from the Middle East (71%) have published in open access journals than from anywhere else, too. Interestingly, while research funder mandates for open access are especially strong in Europe, fewer researchers (56%) there told us that they have published their work under an OA model compared to those in the Middle East and compared to researchers in Central Asia (66%), Africa (65%), or the Asia‐Pacific region (64%). The amount of OA publishing in Europe beat out only the USA, where just 46% reported that they have published their work open access. Demand for more society OA publishing among members seems to be less tied to their local funding environment and more connected to how much open access publishing they have done themselves. This also fits with the response we saw when we asked about society funds to cover Open Access Article Publication Charges (APCs). Researchers in Europe are also less motivated to join a society by the offer of funds to help with APCs, perhaps because finding funds for APCs is not a particular challenge for them. Funding help for APCs would be a big draw for members in Africa and Central Asia, though, which are also the two places learned societies are most likely to find new members. https://www.wiley.com/network/societyleaders/societies/how‐much‐where‐and‐when‐do‐members‐want‐open‐access.

Open access can and should mean more than just being able to download an article without cost. It also means that the information available should be accessible to the reader and not so dense that it is difficult to understand or integrate into the user’s body of knowledge. Articles are often written just for other scholars in a narrow field. How do you make these articles available to those in adjacent fields or to those in a broader scientific landscape of scholars? How do you get the information of science and engineering into communities that do not have access or have barriers to access? Accessible means you can tell me what you did, why you did it and what it means, and you can do so in a way that is understood by a wider space of scholars. https://soundcloud.com/specialissue/episode‐38‐open‐access‐doesnt‐mean‐equal‐access‐what‐does‐it‐mean‐to‐be‐open.

So how are Societies navigating open access? First, the journals must have an impact on scholarship and on people’s lives, secondly the journal must be sustainably funded so we can continue to deliver the research and assure that the Society’s priorities are aligned with Wiley’s. The publishers and societies that have seriously discussed such priorities have done well with open access. There is an emphasis on helping scholars put their information into visuals that better help communicate thoughts and ideas and gives the readers a more accessible version of science. https://www.wiley.com/network/societyleaders/societies/how‐societies‐are‐navigating‐open‐access?elq_mid=48002&elq_cid=12309687&utm_campaign=30355&utm_source=eloquaEmail&utm_medium=email&utm_content=Email%206‐RC‐SOCM‐MS‐XX‐Global‐W26M4‐October%20Newsletter.

Finally, Wiley has a page of links to articles focusing on various topics related to open access publishing and open data. I encourage you to explore this page as you may find an article that will illuminate and challenge you to more creative ideas for communication. https://www.wiley.com/network/researchers/open‐access‐week‐2020‐open‐with‐purpose?elq_mid=48002&elq_cid=12309687&utm_campaign=30355&utm_source=eloquaEmail&utm_medium=email&utm_content=Email%206‐RC‐SOCM‐MS‐XX‐Global‐W26M4‐October%20Newsletter.

